# Ruptured Hydatid Cyst with an Unusual Presentation

**DOI:** 10.1155/2011/730604

**Published:** 2011-08-15

**Authors:** Deepak Puri, Amit Kumar Mandal, Harinder Pal Kaur, Tek Singh Mahant

**Affiliations:** Fortis Hospital Mohali, 193, Phase XI, Mohali, Punjab 160062, India

## Abstract

Ruptured pulmonary hydatid cyst may sometimes cause complications like empyema, bronchopleural fistula, and collapsed lung. These complications may mislead the diagnosis and treatment if prior evidence of cyst has not been documented before rupture. We present a case of a young male who presented with complete collapse of left lung with pyopneumothorax and bronchopleural fistula which was misdiagnosed as pulmonary tuberculosis. He was referred to us from peripheral hospital for pneumonectomy when his condition did not improve after six months of antitubercular chemotherapy and intercostals drainage. On investigation, CT scan revealed significant pleural thickening and massive pneumothorax restricting lung expansion. Decortication of thickened parietal and visceral pleura revealed a ruptured hydatid endocyst, and repair of leaking bronchial openings in floor of probable site of rupture in left upper lobe helped in the complete expansion of the collapsed lung followed by uneventful recovery.

## 1. Introduction

Rupture of the pulmonary hydatid cyst is not very uncommon complication. Rupture can occur either into the pleural cavity or bronchus [1]. Rupture into pleural cavity present as pleural effusion and subsequent complications include infection leading to empyema (in 7.6% cases), pleural thickening, collapsed lung, simple pneumothorax (in 2.4%–6.2% cases) [2] and tension pneumothorax [1], bronchopleural fistula, and large residual cavity. The primary etiology in such patients may be at times missed and patient treated for other more common causes of such complications like pulmonary tuberculosis and may even be subjected to major lung resection or pneumonectomy by anunsuspecting surgeon.

We present one such rare case of a young male with pyopneumothorax and bronchopleural fistula (BPF) misdiagnosed as pulmonary tuberculosis in peripheral centers as this disease is endemic in our country. Later on, when he did not recover after intercostal drainage and 6 months of antitubercular treatment, he was referred to us for pneumonectomy which was actually not required. On exploratory thoracotomy and careful observation, the etiology was found to be actually complications of ruptured hydatid cyst.

## 2. Case Report

**Figure 1:**
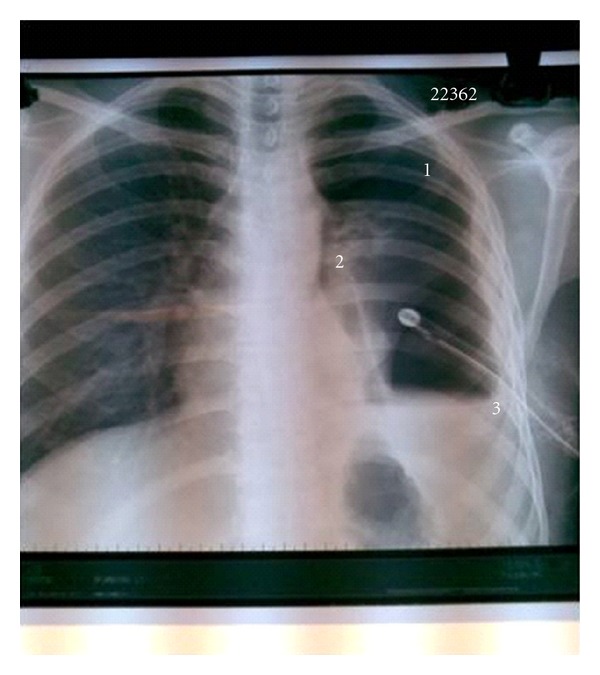
Chest roentgenogram showing massive left pneumothorax (1) with collapsed left lung (2). Intercostal drain is seen in situ with residual pyothorax (3).

**Figure 2:**
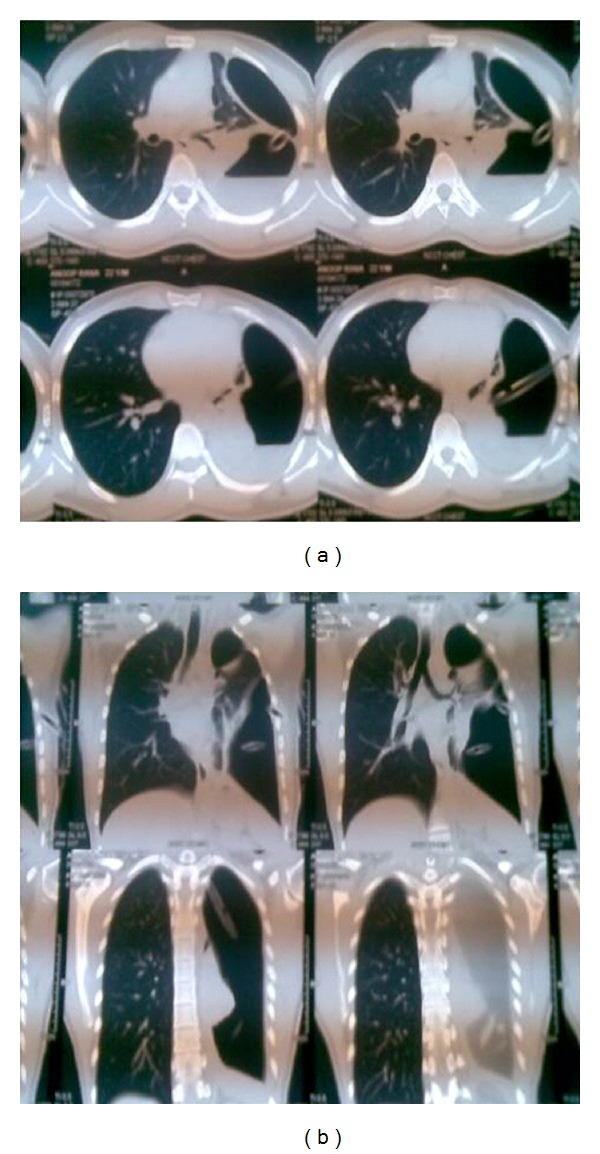
(a) (transverse sections) and (b) (coronal sections) contrast-enhanced computerised tomogram chest showing massive left pneumothorax with near complete collapse left lung and significant pleural thickening with adhesion to chest wall. Intercostal drain with residual pyothorax is also seen.

**Figure 3:**
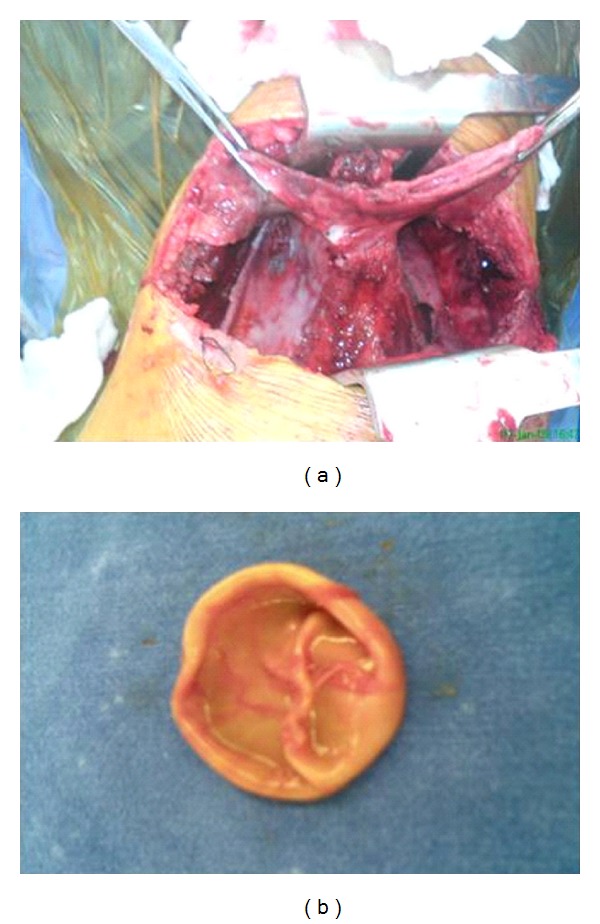
(a) Collapsed left lung with pleural thickening and adhesions and massive air leak preventing expansion. (b) Ruptured endocyst found adherent to left upper lobe.

**Figure 4:**
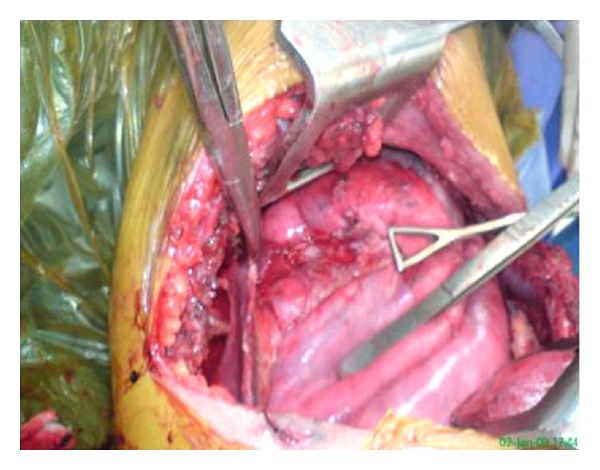
Left lung after decortications with leaking bronchial openings in floor of left upper lobe which were repaired.

A 23-year-old male who had complaints of fever and left-sided chest pain six months before he was referred to us had been diagnosed as tubercular pyopneumothorax with bronchopleural fistula and given antitubercular chemotherapy with intercostal drainage (ICD) at several thoracic centers. He was finally referred to us for pneumonectomy, as his collapsed left lung had failed to expand despite repeated intercostal drain repositioning and standard multidrug antitubercular treatment. On presentation, he was febrile, BP was 120/70, and pulse rate was 96/minute. On chest examination, there was grossly decreased air entry on the left side with amphoric breath sounds tympanitic percussion note due to massive pneumothorax. Left intercostal drain which had been inserted since several months was draining pus which was growing Staphylococcus aureus with severe air leak on inspiration as well as expiration. On CXR, there was large pneumothorax on left side with completely collapsed left lung, while the right lung field was normal (Figure 1). CT chest revealed left massive pneumothorax with near complete collapse of the left lung and overlying grossly thickened pleura (Figure 2). The patient was posted for decortications, and left posterolateral thoracotomy was done. Left pleural cavity was entered and massive air leak with completely collapsed left lung was noticed. About 200 mL of whitish pus was drained from left pleural cavity. Parietal pleura was thickened >1 cm and visceral pleura was approximately 2-3 mm. Thick peel of 1 cm covering the left lung was removed along with thick adhesions, and underlying lung was released. A ruptured hydatid endocyst was found adhered to the thickened pleura overlying left lower lobe (Figures 3(a) and 3(b)). A bronchopleural fistula was seen originating from floor of probable site of ruptured cyst cavity in left upper lobe of lung which was repaired with prolene sutures (Figure 4). The lung expanded fully on table with no significant parenchymal air leak. Thoracotomy was closed in layers and patient was shifted to ICU for elective ventilation. He was extubated within 2 hours, and no post procedure air leak was noticed. The patient was discharged with left ICD, as he had left residual pleural effusion which stopped within 15 days, and the pleural cultures became sterile, so the ICD was removed. In followup after 1 month, 6 months, and 1 year, patient remained asymptomatic, and there was no recurrent effusion or pneumothorax, and CXR was normal.

## 3. Discussion

One of the unusual complications of pulmonary hydatidisosis is rupture which can occur spontaneously when the size reaches 7–10 cm in diameter, secondarily due to an infectious process, trauma to the chest, coughing, or after needle aspiration [3]. The incidence of rupture has been seen to be more frequent in children and reported to be 26.7% in one series [1]. Infection of the cyst can result in abscess with total purulent destruction of the parasite [3]. Rupture of the cyst can cause spillage of the contents to the pleural cavity [1]. Progressive enlargement of hydatid cyst can erode adjacent bronchus which initially does not function as fistula due to pressure of tense cyst obliterating bronchial opening. However, further enlargement results in rupture of cyst into either the bronchus or pleural cavity which presents as pneumothorax or hydropneumothorax. Occasionally, such patients present with allergic manifestation like urticaria, asthma, or anaphylactic shock. 

Most frequent symptoms in cases of rupture are cough and fetid expectoration [1]. Any acute symptom such as cough, hemoptysis, fever, chest pain, vomiting or membrane expectoration, or sudden aggravation in one of these symptoms is indicative of rupture [1]. 

Ruptured infected hydatid cysts are usually difficult to diagnose radiologically as cysts that rupture into pleura are misdiagnosed as empyema [1]. CT scan has been reported to be the most sensitive diagnostic tool [4]. Serological tests like Casoni skin test and Weinberg test are not specific [1] but immunoblot assay has >99% specificity and is highly sensitive [5]. Eosinophilia increases significantly on cyst rupture [6]. However, these investigations are usually not conducted when a patient presents with pyopneumothorax with bronchopleural fistula unless there is a strong suspicion of pulmonary hydatidosis.

In our case, the patient presented with pyopneumothorax and BPF, and there was no suspicion of hydatid etiology; therefore, he was being initially treated as a case of pulmonary tuberculosis for 6 months in peripheral hospital, as tuberculosis is endemic in our country and is the usual cause of empyema with BPF. When six months of antitubercular chemotherapy and repeated intercostal drain repositioning and appropriate antibiotics to treat superadded pyothorax failed, the patient was referred to us for pneumonectomy. However, as CT showed thickened pleura restricting the lung, so decortication of the parietal and visceral pleura was planned by us to release the lung and during the process ruptured hydatid endocyst was discovered. Capitonnage of ruptured cyst floor after BPF repair helped in obliterating the residual cavity and stopping air leak. With careful observation and correct planning of the procedure, correct diagnosis and treatment became possible, pneumonectomy was avoided, and left lung expanded completely after the procedure.

Various postoperative complications can still occur in such cases. Early complications include unexpanded lung (which usually responds to respiratory physiotherapy), persistent air leakage, persistent empyema and wound infection, fistula formation, and seroma formation in residual cyst cavity [7], while late complications include persistent bronchopleural fistula [8]. However, our patient had an uneventful postoperative recovery and remained asymptomatic in a followup of one year.

## 4. Conclusion

Ruptured hydatid cyst can present in an unusual way as pyopneumothorax with BPF. Correct diagnosis can be missed many times and be confused with pulmonary tuberculosis with superadded empyema. Strong clinical suspicion, sharp observation and judicious exploratory thoracotomy, and decortication along with repair of bronchial openings in the floor of the cyst helped in allowing the lung to expand fully, making it possible to salvage the lung which appeared hopelessly affected on preoperative CT chest.

## References

[1] Balci AE, Eren N, Eren S, Ulka R (2002). Ruptured hydatid cysts of the lung in children: clinical review and results of surgery. *Annals of Thoracic Surgery*.

[2] Aribas OK, Kanat F, Gormus N, Turk E (2002). Pleural complications of hydatid disease. *Journal of Thoracic and Cardiovascular Surgery*.

[3] Sadrieh M, Dutz W, Navabpoor MS (1967). Review of 150 cases of hydatid cyst of the lung. *British Journal of Diseases of the Chest*.

[4] Gulalp B, Koseoglu Z, Toprak N (2007). Ruptured hydatid cyst following minimal trauma and few signs on presentation. *Netherlands Journal of Medicine*.

[5] Basavana GH, Siddesh G, Jayaraj BS, Krishnan MG (2007). Ruptured hydatid cyst of lung. *Journal of Association of Physicians of India*.

[6] Ozvaran MK, Unver E, Uskul TB (2000). An evaluation of diagnosis and treatment of pulmonary hydatid cyst in patients over 50 years old. *Turkish Respiratory Journal*.

[7] Pirmoazen N, Saidi F, Ahmadi ZH, Firouzi F (2004). The surgical management of Complicated pulmonary hydatid cysts. *Medical Journal of the Islamic Republic of Iran*.

[8] Shalabi RIY, Ayed AK, Morsi A (2002). 15 years in surgical management of pulmonary hydatidosis. *Annals of Thoracic and Cardiovascular Surgery*.

